# Influence of cervical cerclage interventions upon the incidence of neonatal death: a retrospective study comparing prophylactic versus rescue cerclages

**Published:** 2018-03

**Authors:** A Wafi, G Faron, J Parra, L Gucciardo

**Affiliations:** Department of Obstetrics and Prenatal Medicine, Universitair Ziekenhuis Brussel, Vrije Universiteit Brussel, Brussels, Belgium; Faculty of Medicine and Pharmacy, Department of Biostatistics and Medical Informatics, Vrije Universiteit Brussel, Brussels, Belgium.

**Keywords:** Rescue cerclage, prophylactic cerclage, fetal outcome, cervical insufficiency, cerclage complications

## Abstract

**Objective:**

The purpose of this study was to compare the efficacy of prophylactic and rescue cervical cerclages for pregnant patients with an incompetent cervix, and to assess the neonatal outcomes of both clinical conditions.

**Methods:**

This was a retrospective observational study of all women who had an elective or rescue cerclage between January 2008 and December 2016 in our institution. Prophylactic cerclage was defined as a cerclage before 16 weeks of gestation, while rescue cerclages were performed between 16 and 23 weeks of gestation.

**Results:**

In total, we analyzed the outcomes of 212 cervical interventions; 71% of the recruited patients experienced prophylactic cerclage, while 29% underwent rescue cerclage. Most of the patients delivered vaginally (70%) and were able to leave the hospital with a healthy newborn (78%). The mean pregnancy prolongation time after cerclage in the prophylactic and rescue groups were 21 weeks and 10 weeks, respectively.

**Conclusion:**

Prophylactic cerclage interventions are most likely to be associated with a reduction of fetal demise because of the correlation between fetal prognosis and the gestational age at which cerclage is performed. Once the diagnosis of cervical insufficiency is confirmed, cerclage should be recommended as this will help to prolong the pregnancy.

## Introduction

While cervical insufficiency is known to involve a progressive softening and shortening of the cervix, the pathogenesis of this condition remains unclear (Brewstera and Walker, 2006). The prevalence of this condition is estimated to be less than 1% (Brown et al., 2013), but remains a major health issue for patients experiencing recurrent late miscarriages or early preterm births (ACOG Practice Bulletin. Cervical insufficiency, 2003). Indeed, preterm birth, defined as delivery prior to 37 weeks of gestation, is still one of the principal causes of perinatal morbidity and mortality worldwide (5–12% incidence) (Harris-Requejo and Merialdi, 2010). Neonatal prognosis is directly dependent upon the gestational age at birth; therefore, once a diagnosis of cervical insufficiency has been established, and provided that there is no maternal contra-indication to maintain the pregnancy, all the available options to prolong the pregnancy should be discussed with the patients (MRC/RCOG Working Party on Cervical Cerclage, 1993).

In 1955, Shirodkar introduced cervical cerclage as a surgical technique to restore cervical function. In 1957, McDonald developed a simpler procedure, a surgical intervention which rapidly became the gold-standard for the treatment of patients with cervical insufficiency. The Shirodkar suture is a trans-vaginal purse string suture inserted after bladder mobilization above the level of the cardinal ligament, while the McDonald suture is inserted lower at the cervicovaginal junction, but without bladder mobilization (Shirodkar, 1955; McDonald, 1957). Cervical cerclage was initially introduced for two main indications: the prevention of second trimester losses for pregnant patients with painless shortening of the cervix (rescue cerclage) or the treatment of recurrent second trimester late miscarriages and/or preterm deliveries (prophylactic cerclage) (Shirodkar, 1955; McDonald, 1957). A few years ago, the use of transvaginal echography (TVE) to evaluate cervical length was highlighted as a useful marker with which to quantify the risk of preterm delivery (Berghella et al., 2013; Feingold et al., 1984). The potential benefits of cerclage indications, based upon abnormal cervical length as measured by TVE, was evaluated in a recent meta-analysis of randomized clinical trials. Cerclage interventions were correlated with significant reductions in preterm birth and perinatal morbidity when compared with non-surgical managements (Berghella et al., 2013).

According to the available literature, the best results are obtained for prophylactic cerclage (Liddiard et al., 2011; Kurup and Goldkrand, 1999; Wu et al., 1996). However, the benefits of rescue cerclage, in terms of prolonging pregnancy, appear to be more limited (Kurup and Goldkrand, 1999; Ciancimino et al., 2015; Daskalakis et al., 2006; Gundabattula et al., 2013; Zhu et al., 2015). An important limiting factor which needs to be highlighted is the presence of a subclinical or obvious chorioamnionitis, which remains a predominant issue for patients with cervical dilation, with an incidence of 9%–33% for patients with bulging membranes. In the same study, prophylactic cerclage was associated with a reduced incidence of chorioamnionitis (1%–7.7%) (Harger, 2002).

The present study aimed to identify and compare the efficacy of these two types of cervical interventions to prolong pregnancy and to assess neonatal outcomes in both clinical conditions.

## Materials and methods

This retrospective study was conducted at the Department of Obstetrics and Prenatal Medicine, University Hospital UZ, in Brussels. Data used for this retrospective analysis were extracted using medical records of all patients who received cerclage in our institution between January 2008 and December 2016. We extracted a range of information from patient medical records and delivery registration, including parity, gestational age at suture insertion, gestational age at delivery, mode of delivery, smoking habits, obstetrical detailed history, number of stitches, and pregnancy outcomes. First trimester fetal ultrasound was used to define accurate gestational age and to exclude structural and chromosomal major anomalies.

Cervical insufficiency was defined as followed: (1) if the patient had a previous history of recurrent (>2) second trimester pregnancy losses and/or preterm deliveries, and/or (2) if a shortening of the cervical length (< 15 mm) was visualized at the endovaginal ultrasound evaluation at 20-24 weeks, or (3) when the patient presented with a dilated cervix and membranes bulging in the vagina with no signs of labor, infections, or heavy vaginal bleeding. Prophylactic cerclage was proposed and performed at the end of the first trimester (10–15 weeks) for non-urgent indications based upon the patient’s obstetric history (recurrent second mid-trimester miscarriages and/or preterm deliveries).

Rescue cerclage was defined as insertion of cervical sutures when membranes were bulging through the cervical canal or shortening of the cervical length was visualized during endovaginal ultrasound.

These interventions were performed only after exclusion of active labor or significant inflammation (negative C-reactive protein, no significant neutrophilia). When the patient was colonized with Ureaplasma spp, she first received clindamycin vaginal cream before and after the procedure. All cervical stitching procedures were performed under 24 weeks of gestational age all according to the McDonald’s technique using ETHIBOND EXCEL® 6-0 (Ethicon, Somerville, NJ). All patients received also vaginal or oral micronized progesterone until 36 weeks (or until the delivery).

Cerclages were removed at 36 weeks of gestation unless the patient presented with premature rupture of the membranes or progressive premature labor. Preterm ruptures of the membranes (PPROM) after 24 weeks were managed with antibiotics and fetal lung maturation according to the hospital policy. Counselling for patients with PPROM <24 weeks, included options for expectant or elective termination of the pregnancy.

The log-rank test was used to compare survival rates in different cerclage groups and a validated Cox proportional hazard was used to account for different confounding variables. Cox regression was validated by checking proportional odds assumption and fit statistics using Cox-Snell residuals (Grambsch and Therneau, 1994; Collett, 1994). Differences in groups of nominal variables were analyzed using the Chi-square test. Ordinal variables were analyzed by the Wilcoxon-Mann-Whitney test (R Core Team, 2015). All data were analyzed with R software (version 3.1.3) and survival analysis was performed using the R “survival” package (Therneau, 2010; 2015).

## Results

In total, 212 consecutive cervical interventions were performed during the study period. The observed incidence of cerclage was 1.2% of all deliveries (n=18,075). Amongst patients treated with a cerclage, 30% (n=64) in this selected high-risk-group were pregnant following in vitro fertilization (IVF) treatment and 10% (n=21) were twin pregnancies. Out of these 212 procedures, 77% (n=163) involved patients receiving cerclage for the first time, while 21% (n=44) were patients receiving cerclage for the second time. Only 2% (n=5) of patients were receiving cerclage for the third time.

The clinical characteristics of the study population are shown in Table I. Most of the patients recruited were multigravida (90%; n=191, including 53 nulliparous), only 16% (n=10) of the rescue cerclage group were primigravida with a mean cerclage at 20 weeks, 74% (n=158) of the patients had a medical history of one previous late miscarriage, while 42% (n=89) reported more than one previous late miscarriage.

**Table I:** Clinical characteristics of the study populations.

Variable	Total population (n = 212)	Patients with elective cerclage (n=151)	Patients with rescue cerclage (n=61)	p-value
Spontaneous pregnancy	148 (70%)	110 (73%)	38 (62%)	0.17
IVF pregnancy	64 (30%)	41 (27%)	23 (38%)	0.09
Singleton	191 (90%)	142 (94%)	49 (80%)	0.005
Twin	21 (10%)	9 (6%)	12 (20%)	0.001

IVF= in vitro fertilization N= number of cases

Nearly half of the recorded deliveries following cerclage intervention occurred between 38 and 41 weeks of gestation (47%, n=100). The general incidence of complications, such as chorioamnionitis or PPROM following cerclage intervention was 40% (n=84) with a significantly higher incidence within the group of patients with a rescue cerclage when compared with the group of prophylactic interventions (respectively 61% and 31%, P=0.01).

Tables II show the pregnancy characteristics and outcomes for patients following live birth compared to patients experiencing fetal demise. Cerclage intervention was performed at a mean gestational age of 14 weeks (range: 10–23) for patients from the live birth group compared to 16 weeks (range: 11–22) for the fetal demise group (P=0.01). Mean pregnancy prolongation following a cervical stitching procedure was 22 weeks in the live birth group compared to only 4 weeks in the fetal demise group.

**Table II:** Pregnancy characteristics and outcomes according to pregnancy issue.

Variable	Live birth (n=165)	Fetal demise (47)	P-value
Mean term at cerclage (in weeks, range)	14 (10-23)	16 (11-22)	
Cerclage			0.036
Elective	126	25	
Rescue	39	22	
Singleton	152	39	0.11
Twin	13	8	0.03
Conisation	18	4	0.8
One Stitch	42	6	0.10
Two Stiches	122	41	0.08
Mean term at delivery (in weeks)	38	20	
Complications			0.0001
Yes	37	47	
No	128	0	
Weight (Mean)	2872g	428g	
Prolongation (Mean, In weeks)	22	4	

N= number of cases

### Prophylactic cerclage

For 151 patients, cerclage was inserted prior to 16 weeks of gestation. Different issues of these pregnancies are summarized in Table III. The mean time interval between cervical suture and delivery was 21 weeks (range: 1–29) with a survival rate of 83% (n=126). For the majority of the registered patients (75%, n=114), two cervical stitches were inserted during surgical intervention. These cases resulted in 81% live births (n=92) and 19% fetal demise (n=22) with a mean pregnancy prolongation of 20±9.2 weeks. On the other hand, 24% (n=36) of these prophylactic cerclage procedures were performed using only one stitch with no significant difference in terms of live birth rate (92%, n=33) or fetal demise (8%, n=3) compared to the two stiches procedure (P=0.489). One patient with a history of late miscarriage received a triple stitch cervical cerclage at 13 weeks of gestation and finally delivered at 39 weeks. Nine pairs of twins were included in the group of prophylactic cerclages, for which the cerclage procedure with two stitches was performed and resulted in 56% live births (n=5) and 44% fetal demises (n=4).

**Table III:** Comparison of outcomes according to the indication of cerclage.

Variable	Total population (n = 212)	Patients with elective cerclage (n=151)	Patients with rescue cerclage (n=61)	P-value
Mean term at cerclage (in weeks)	15	13	19	
Prolongation (in weeks)	18	21	10	
Mean gestational age at delivery (in weeks)	33	34	29	
Vaginal delivery	149 (70%)	104 (69%)	45 (73,8%)	0.14
C-Section	63 (30%)	47 (31%)	16 (26,2%)	0.14
Live birth	165 (78%)	126 (83%)	39 (63,9%)	0.37
Single stitch	48 (22,6%)	36 (24%)	12 (19,7%)	0.64
Two stitches	163 (78%)	114 (75,5%)	49 (80,3%)	0.35
Complications	84 (40%)	47 (31%)	37 (60,7%)	0.0001

N= number of cases

### Rescue cerclage

Sixty-one patients were included in this group and were generally considered as requiring urgent obstetric management. These patients presented with cervical dilation and intact membranes but without evidence of labor or infection. The mean time interval between cervical suture and delivery was 10 weeks (range: 0–24) with a 64% (n=39) live birth rate and 36% (n=22) fetal demises. Twelve sets of twin pregnancies were included; all of these patients received the cerclage procedure with two stitches and demonstrated a live birth rate of 67% (n=8) and 33% fetal demises (n=4).

Complete results are summarized in Table III. The general incidence of fetal demises within this group was relatively higher compared to prophylactic cerclage (34% versus 17%). For the majority of the registered patients, 80% (n=49) received two cervical stitches, whereas 20% (n=12) of these rescue cerclage procedures were performed using only one stitch.

### Comparison of neonatal outcomes according to the indication of cerclage

**Figure 1:**
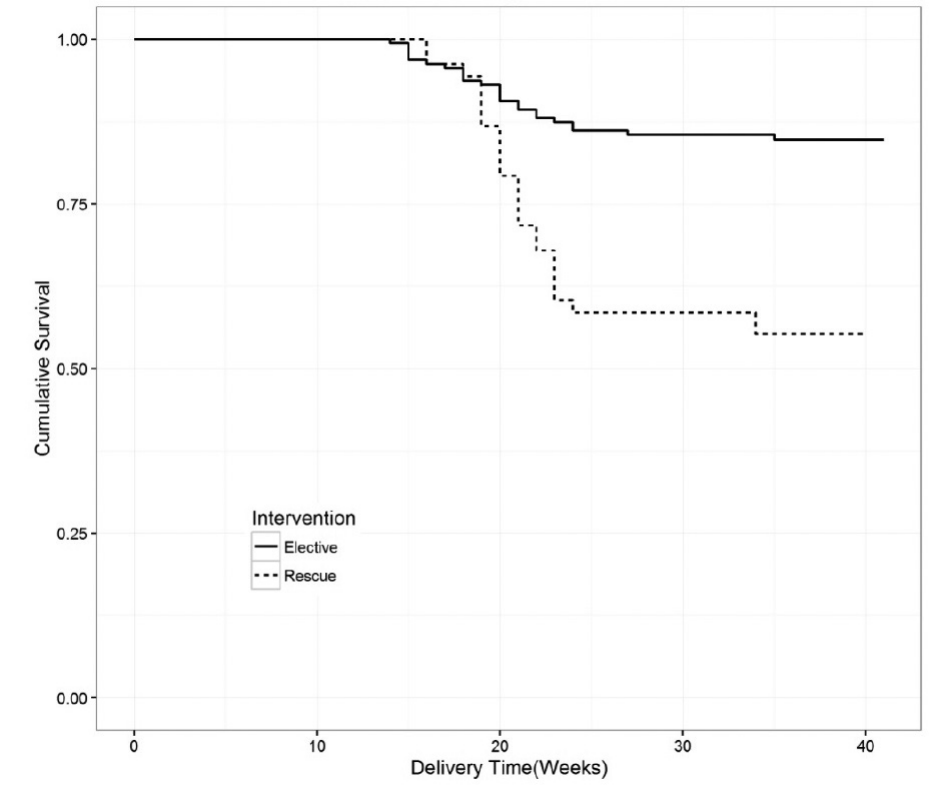
— Kaplan Meier Curve with survival of newborns by time.

We observed significant differences in survival rates among neonates according to the type (prophylactic vs rescue) cervical cerclage (Fig.1). The hazard ratio of neonatal death in the group of patients receiving rescue cerclage was approximately three times higher than in the group receiving prophylactic cerclage (HR: 3.3 [1.8, 6.1]). Multivariate analysis was performed and confirmed the same observation with a hazard ratio of neonatal death, which was three times higher in the group of patients receiving rescue cerclage (HR: 2.8 [1.3, 6.3]) than for the patients receiving prophylactic cerclage.

**Figure 2:**
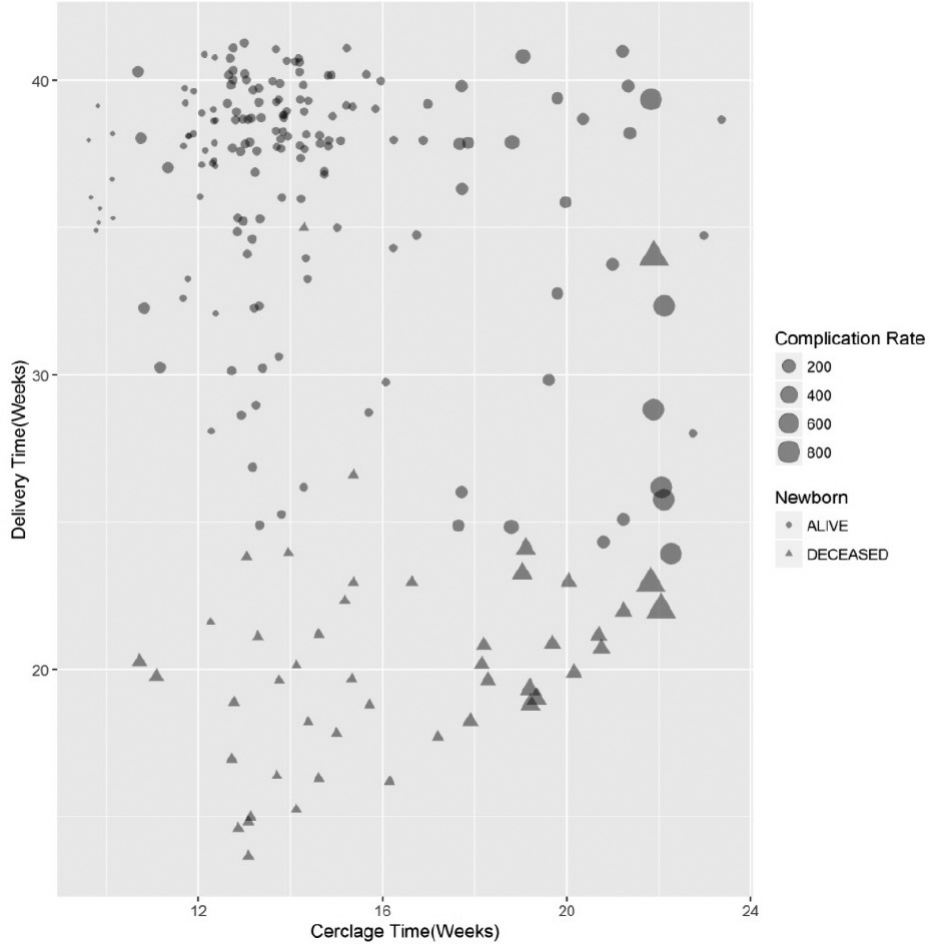
— Jittered scatterplot of newborn survival data according to indication of cerclage (elective versus rescue). *Complication rate per week

In addition, we investigated the correlation between neonatal outcomes and the gestational age at which the cerclage was performed. The scatterplot (Figure 2) indicates that cerclage at weeks 16 and 17 is associated with the least incidence of death, and it is evident that the risk of complications and death increase when the cerclage is performed after 18 weeks.

Even with the small number of events after 20 weeks, we can notice in Figure 2 that complications and fetal demise events are much higher in patients who have had their cerclage after 20 weeks. In contrast, we observe a much lower complication and fetal demise events in the group of patients having had their cerclage before 14 weeks. Comparison of neonatal outcomes according to history indicated cerclage

We conducted a comparison between patients having had both type of procedures in 2 different pregnancies: generally a rescue cerclage during the first one and a prophylactic procedure during the subsequent pregnancy. Out 44 concerned patients, we excluded 12 of them because they have had their rescue cerclage in other institutions. Table IV summarizes the pregnancy characteristics and outcomes for this subgroup of patients.

**Table IV:** Pregnancy characteristics and outcomes for patients having had cerclage during at least 2 consecutive pregnancies.

Variable	First pregnancy with cerclage (n=30)	Subsequent pregnancy with cerclage (n=30)	P-value
Mean term at cerclage (in weeks, range)	17 (12;22)	13 (10;17)	
Cerclage			0.0001
Elective	12	28	
Rescue	18	2	
Singleton	25	29	
Twin	5	1	
One stitch	11	9	
Mean term at delivery (in weeks)	28 (16;41)	34 (16;41)	
Complications			0.0001
Yes	19	9	
No	11	21	
Weight (mean)	1707g	2596g	
Prolongation (mean, In weeks)	11	22	
Live birth	15	26	0.0001
Fetal demise	15	4	

N= number of cases

Cerclage intervention was performed at a mean gestational age of 13 weeks (range: 10-17) for patients from the repeated cerclage group compared to 17 weeks (range: 12–22) for the first procedure group (P=0.01). Comparing the pregnancy outcomes, we observed a significant difference in the survival rate among neonates born in the repeated cerclage (28/30 prophylactic) group: 87 % versus 50% in the first procedure (18/30 rescue) group. This was associated in this last one by a higher complication rate (63%, 19/30) compared to the repeated one (30%, 9/30), which can explain its higher perinatal morbidity.

## Discussion

The aim of this study was to evaluate neonatal outcomes among a population of 212 pregnant patients treated with prophylactic or rescue cervical cerclage. The incidences of cervical insufficiency among our group of patients were comparable to previous published research (1%) (Brown et al., 2013; Romero et al., 2006; Osemwenkha and Osaikhuwuomwan, 2014). The identification of patients with a history of previous late miscarriages remains a cornerstone to prevent recurrent pregnancy losses in patients with the potential diagnosis of an incompetent cervix (Romero et al., 2006; Simcox et al., 2007). This was confirmed by our observations of a strong correlation between the indication of a cervical stitching procedure and the history of late miscarriage; 74% of all registered interventions were motivated by a history of at least two late fetal demises. Clinical tools have been developed to improve the selection of candidates for cervical cerclage interventions. Heath et al. showed that the risk of premature delivery was related to the cervical length, as measured by vaginal echography (Heath et al., 1998). The implementation of TVE in clinical practice changed prenatal follow-up programs and led to the inclusion of TVE measurements of cervical length as a useful screening tool for the early detection of cervical insufficiency (Berghella et al., 2013; Feingold et al., 1984). The small number of events in the rescue group described herein unfortunately prevented us from using statistical analysis to determine the best timing to perform cerclage. Nevertheless, we were able to create a scatterplot which indicated that cerclage at 16–17 weeks had the least incidence of death. Consequently, we recommend that physicians perform TVE cervical length measurements around 16–17 weeks of gestational age for patients with a high-risk of late miscarriage or preterm birth.

However, surgical habits are not always evidence-based. In many obstetric units, the two-stitch cervical cerclage procedure is the preferred option, based on a subjective impression of better outcomes. In our unit, we used one or two stitches at random. Nevertheless, our results confirmed previous published research showing that there is no significant difference in term of survival between the placement of one or two stitches when neonatal outcome is considered (Woensdregt et al., 2008; Park et al., 2012; Giraldo-Isaza et al., 2013). None of the earlier studies have reported the benefit of one technique over the other (ACOG Practice Bulletin. Cervical insufficiency, 2003; MRC/RCOG Working Party on Cervical Cerclage, 1993), despite the fact that most studies appear to show superior results for early cerclage (ACOG Practice Bulletin. Cervical insufficiency, 2003; Liddiard et al., 2011; Kurup and Goldkrand, 1999; Wu et al., 1996). Without clear evidence that two-stitch cervical cerclage is associated with significantly improved outcomes, we recommend the use of a less invasive cervical cerclage procedure involving only one stitch.

In 2003, Althuisius et al., (2003) published a prospective study including 23 patients with incompetent cervix; 13 of these patients were randomly allocated to a rescue cerclage group and 10 were restricted to a bed rest-only group. Results showed that cerclage interventions were associated with significantly longer pregnancy prolongations (54 and 20 days respectively). Daskalakis et al., (2006) arrived at the same conclusion in an observational prospective study; 46 patients with a short cervix (<15 mm) were recruited, 29 were treated by rescue cerclage and 17 refused rescue cerclage and were included in a bed rest expectative follow-up group. This study reported the improved prolongation of pregnancy and neonatal survival when a cervical stitching intervention was performed compared to bed rest. Several other studies published similar results showing evidence that rescue cervical cerclage could prolong pregnancy by 7 to 12 weeks (McDonald, 1957; Liddiard et al., 2011; Kurup and Goldkrand, 1999; Woensdregt et al., 2008). Our present data confirmed these reported observations in terms of the extension of pregnancy following rescue cerclage intervention (10 weeks). These results further imply that rescue cerclage is a more favorable approach, which can lead to increased odds for the delivery of a viable infant.

The observed pregnancy prolongations for the patients of our study who were managed with a prophylactic cerclage (21 weeks) are in line with previously reported results (20–22 weeks) (Liddiard et al., 2011; Kurup and Goldkrand, 1999; Khan et al ., 2012).

Outcomes of patients from the prophylactic group were much better than corresponding results reported for patients treated with a rescue cerclage, thus confirming observations made by several previous publications (Liddiard et al., 2011; Kurup and Goldkrand, 1999; Wu et al., 1996; Harger, 2002). The number of patients who were able to leave the hospital with a healthy baby was significantly higher after prophylactic intervention than after rescue cerclage (83% and 64%, respectively). Furthermore, the incidences of PPROM and chorioamnionitis were significantly increased in the rescue cerclage group compared to the group receiving prophylactic interventions. This could be explained by exposure of amniotic membranes to the vaginal microbial environment, and the consequence of this upon sub-clinical infections. Sub-clinical infection is considered to represent a major problem which should be adequately addressed before and after a cervical stitching intervention. MacDougall and Siddle (1991) recommended bacteriological assessment prior to insertion of the cerclage. More recent publications showed that the introduction of MIAC (microbial invasion of the amniotic cavity) testing with intra-amnionic Gram stain and/or glucose tests, can help with the diagnosis of subclinical chorioamnionitis. When these infected patients are excluded from cerclage, the likelihood of prolonging pregnancy is increased (Lisonkova et al., 2014; Mays et al., 2000). For the entire patient cohort included in our study, cerclage was only performed if active signs of labor or significant inflammation (negative CRP with no increasing trend of WBC counts) were excluded. Cervical stitches were removed if the patient had infectious symptoms, with a rise in CRP, despite the use of prophylactic antibiotics.

In conclusion, the retrospective design and the consequent restriction of data standardization have to be considered as limitations of this study. Nevertheless, our findings are consistent with the existing literature. Our results support the use of cerclage interventions to prolong pregnancy with a consequent reduction of fetal losses and neonatal deaths. The majority of our patients managed with an elective or a rescue cerclage delivered a healthy live-born baby. Cerclage interventions allowed more than half of the patients from our rescue group to extend their pregnancy from pre-viability to prematurity. Therefore, cerclage remains probably the best option for patients with cervical insufficiency.
